# Supplementary material for: Corneal confocal microscopy identifies greater corneal nerve damage in patients with a recurrent compared to first ischemic stroke
Source: PLoS One. 2020 Apr 22;15(4):e0231987. doi: 10.1371/journal.pone.0231987 (PMC7176137; doi:10.1371/journal.pone.0231987)
Supplement: Attachment — Submitted filename: Point to Point Response to Recurrent Paper.docx [file pone.0231987.s001.docx]

Dear Dr. Masaki Mogi,

We are grateful to you and the reviewers for providing constructive comments to help us to improve our manuscript. We have made a point by point reply to the comments and made changes (in red) in the manuscript.

**Reviewer # 1:** This paper provides new data suggesting that corneal confocal microscopy demonstrates greater corneal nerve damage in patients with recurrent stroke compared to the patients with first stroke and healthy control subjects. The study protocol is correct, the used technology is innovative, and the paper is well written.

**Thank you for your positive comments.**

Reviewer #1: Below are my minor recommendations:

- Introduction (Page 3, Line 4): The word “smoking” has been repeated twice in the same sentence.

**Thank you, the word “smoking” has been deleted.**

- Introduction (Page 3, Paragraph 2, Line 1): Please edit as “… multiple white matter …”

**Thank you we have edited ‘’…multiple whiter matter...” to “… multiple white matter …”**

- Materials and Methods (Page 5, Lines 2,3): “The size of each 2-dimensional image produced is 384x384 μm…” Please edit as “384x384 pixels” or “400x400 µm”.

**Thank you, “The size of each 2-dimensional image produced is 384x384 μm…” has been edited as “384x384 pixels”.**

- Materials and Methods (Page 4, Line 5): Please use commas rather than semicolons.

**Thank you, semicolons have been replaced with commas.**

**Reviewer #2:** The authors describe a possible prognostic utility of corneal nerve fiber loss in patients with recurrent stroke.
The paper is overall well-written, the methods and results are presented in a concise and easy-to-follow way. The findings are of interest, although the larger studies are warranted to establish the clinical utility of corneal confocal microscopy in ischemic stroke, as the authors also pointed out in the discussion. There are couple of points that are missing or should be described more clearly:

**Thank you for your positive comments**

1. Materials and Methods:
How was the healthy control subject defined.

Were there any inclusion/ exclusion criteria except no history of stroke? Given the age of the participants, I would assume that they were not all completely without any health problem or medication? Please, specify this.

**Thank you for your comments. The healthy controls had no known history of diabetes, other cause of peripheral neuropathy or previous stroke and they were not currently on any medication for any other chronic disease. Also, as can be seen from table 1 they had normal BMI, systolic and diastolic blood pressure, lipid profile and HbA1c.**

2. Discussion

The corneal nerve fiber loss has largely been studied in diabetic patients. It was hypothesized in these patients that the corneal nerve fiber damage may reflect the damage of peripheral nerve system mainly, and the retinal nerve fiber loss (assessed e.g. by optical coherence tomography) may reflect the CNS damage better. Please, discuss a possible etiopathogenetic mechanism that advocates the notion that corneal nerves status is closely tied to the risk of recurrent stroke more clearly.

**Thank you. We believe that both stroke and corneal nerve loss have common risk factors. Indeed, we have set out the rationale for undertaking CCM in this study in the introduction (Page 3, lines 19-21 and page 4 lines 1-6:**

‘More recently we have shown a significant loss of corneal nerves in patients with TIA [31] and acute ischemic stroke [32-34], which was associated with elevated triglycerides and HbA_1c_. Vascular risk factors including dysglycemia and dyslipidemia [35] and hypertension [36] are associated with corneal nerve loss and an improvement in blood pressure, lipids, HbA_1c_ [37] and glucose tolerance [38] are associated with an improvement in corneal nerve morphology.

Given that there are shared risk factors for stroke and corneal nerve loss, we hypothesized that patients with recurrent ischemic stroke will demonstrate greater corneal nerve abnormality compared to those with first ischemic stroke, reflecting the greater overall exposure to the risk factors for stroke.’

**And we have highlighted in discussion Page 10 line 2-6, how alterations in corneal nerves may be linked to recurrent stroke:**

‘In the present study, despite age, BMI, HbA_1c_, lipids, BP and use of medications to treat blood pressure and lipids being comparable between those with recurrent stroke and 1^st^ stroke, there was greater corneal nerve damage in patients with recurrent compared to 1^st^ stroke. This suggests that corneal nerve loss may reflect the cumulative effect of known vascular risk factors and unknown risk factors for stroke and act as a surrogate marker for the risk of stroke and recurrent stroke.’
